# Expectations for future care provision in a population-based cohort of baby-boomers

**DOI:** 10.1016/j.maturitas.2018.08.004

**Published:** 2018-10

**Authors:** Mai Stafford, Diana Kuh

**Affiliations:** a The Health Foundation, 90 Long Acre, London, WC2A 9RA, UK; b MRC Unit for Lifelong Health and Ageing at UCL, 33 Bedford Place, London, WC1B 5JU, UK

**Keywords:** Personal care, Care preferences, Social support, Life course

## Abstract

**Objective:**

We describe sources expected to provide for future care needs among baby-boomers in their late sixties and examine how expectations vary according to earlier health and social experiences. We hypothesised that greater integration in social relationships across adulthood is associated with greater expectation of informal care, and that greater morbidity over a longer time period is associated with greater expectation of formal care.

**Method:**

The MRC National Survey of Health and Development, a population-based birth cohort study set in mainland Britain, provided data on care expectations for 2135 participants aged 68–69. The outcome was who, besides the partner or spouse, is expected to provide for the future care needs, coded as adult children, other relatives, friends/neighbours, paid/professional care, or no one. Adult children were taken as the reference category and the latter two categories were combined as ‘formal care’ in the multiple regression analysis.

**Results:**

91% had an adult child, of whom 74% expected them to provide care if needed, and 11% expected formal care. The latter rose to 33% of those with no adult children. Geographical distance to adult children (over 25 miles) was strongly correlated with expectations but, independently of this, lack of someone to help in a crisis from midlife onwards and low social contact were associated with expecting formal care. Expectations did not differ by number of chronic conditions, functional limitations or longstanding illness from age 60 + .

**Conclusion:**

Those lacking social relationships in midlife onwards and those living further from adult children are more likely to expect formal help with their future care needs. As personal care needs are projected to rise with population ageing and families are increasingly expected to provide for these needs, initiatives to remove barriers to smaller distances between ageing parents and their children and to support and maintain high-quality family relationships across the life course should be considered.

## Highlights

• Over 2000 baby-boomers were asked who they expect will provide their social care, should they need it in the future.
• 3 in 4 people with a living child expect them to meet any future care needs.
• 1 in 5 expect their needs to be met by a friend or by formal care services, rising to 3 in 4 people without a child.
• Low mid-life social contact is linked with greater expectation of formal care.
• Chronic conditions and functional limitations are not related to expectations.

## 1 Introduction

The challenges in meeting current and future social care needs and expectations are being widely discussed [1]. Continued rises in the need for long-term care are projected [2,3]. In Britain and other Western societies, the majority of older people needing help and care with activities of daily living receive this informally from family either from their spouse or partner or their adult children [4,5]. As life expectancy continues to increase at the oldest ages, more adult children with parents needing care may be already at retirement age themselves and less able to help. Care by adult children may or may not align with the preferences and expectations older people, and future generations of older people, have for their own care needs. Preferences and expectations may be changing in light of societal trends relevant for care provision including smaller family sizes and increasing childlessness, an increase in women’s paid employment, and increasing residential mobility [[6], [7], [8]]. Understanding the expectations that the baby-boomer generation has for their future care needs may help with planning and identifying groups at risk of not having their needs met in the expected way.

Studies have categorised preferences for informal, formal or mixed (formal plus informal) support and found they depend on socioeconomic and demographic factors [9]. Higher educational attainment is associated with greater preference for formal care [10,11], a pattern which is reflected in actual receipt of formal support [12,13]. Being married and having more contact with relatives is associated with a greater preference for informal or mixed support compared to formal support [9] whereas marital dissolution is associated with fewer transfers to elderly parents in midlife [14,15] and may have long-term consequences for later informal care.

Most previous quantitative studies are cross-sectional and considered only proximal factors. The impact of earlier health and social experiences on later life expectations for different types of care has received insufficient attention. Care-giving and receiving are a fundamental part of social relationships across the life course [16] so earlier support exchanges may influence the extent to which a person feels able to depend on an informal support network in older age. Chronic exposure to poor health may also affect care preferences and expectations [9] whereas the literature on the role of current activity limitations is inconsistent [9,11,17,18]. The aim of the current study was to describe the main sources expected to provide help with daily activities in a population-based baby-boomer cohort approaching their seventies. Arguably, expectations are based on a pragmatic assessment of a person’s current and future circumstances and social care needs but they are hypothetical in nature. We examine how these expectations vary according to socioeconomic position, social networks, health, and care-giving experience in middle to late adulthood. We hypothesised that i) a more advantaged socioeconomic position would be associated with greater expectation that care needs will be met formally; ii) greater integration in social relationships across adulthood would be associated with greater expectation of informal care, and iii) greater morbidity over a longer time period would be associated with greater expectation that needs will be met formally (by a paid professional) because informal networks may not be able to provide intensive help.

## 2 Data & methods

The MRC National Survey of Health and Development is a representative sample of 2815 men and 2547 women who were born in England, Scotland and Wales in one week in March 1946. The 24th data collection was conducted between 2014 and 2015 when study members were aged 68–69 years. Of the 2816 people in the target sample living in mainland Britain, 2370 (84.2%) completed a postal questionnaire. Of the remaining 2546 (47%) study members: 957 (18%) had already died, 620 (12%) had previously withdrawn permanently, 574 (11%) lived abroad, and 395 (7%) had been untraceable for more than 5 years [19]. Study members found to be still living in Great Britain (n = 2698) were invited to have a home visit by a research nurse: 2149 (79.7%) completed this. For this data collection, we obtained ethical approval from the NRES Queen Square REC (14/LO/1073) and Scotland A REC (14/SS/1009). The main adult sweeps prior to this were conducted when study members were 26, 33, 43, 53, and 60–64 years.

### 2.1 Expectations for future care

During the home visit, participants were asked who would be most likely to provide help in the event that they (and their spouse or partner) needed help with daily activities because of sickness, frailty or disability. Responses were coded as: daughter or son; other family member; friend, neighbour or voluntary worker; paid professional help. Ten participants who were unable to nominate a person were combined with those who nominated paid professional help in the analysis. The spouse or partner was not permitted as a response option because this would likely be collinear with current partnership status [20,21]. Participants were asked to nominate only one source and where two or more were nominated (n = 737), these were coded according to the priority order listed above (e.g. if both daughter and other family member were nominated, the response was coded as “daughter” for analysis).

Information on family factors, socioeconomic position, characteristics of the social network, care-giving experience, and health was collected at several ages.

#### 2.1.1 Family factors

Marital status and marital transitions were captured at each adult sweep. Geographical proximity to the nearest adult child was captured at age 68–69.

#### 2.1.2 Socioeconomic position

We included occupation of the head of the household at age 53 (the most recently available data preceding changes related to retirement), coded using the Registrar General’s classification. We also included highest educational qualification attained by age 26 (when most participants in this cohort had finished full-time study).

#### 2.1.3 Characteristics of the social network

These included both quality and quantity of social contact. At ages 68–69 and 60–64, study members reported the frequency of visits with family not living in the same household and with friends. We combined these to create a cumulative social contact score with high values indicating greatest contact. At ages 60–64, 53 and 43, they reported whether they had any friends, neighbours or relatives who would help if a problem or crisis came up. These were also combined to create a cumulative score. At age 43, study members were asked whether they were emotionally close to their surviving parents.

#### 2.1.4 Care-giving experience

This included hours of care provided for someone frail or with a disability within or outside the home at ages 68–69 and 60–64. These were combined to classify participants into those who provided no care, those who provided 20+ hours of care at both ages, and those providing intermediate levels of care. At age 43, we identified study members who provided at least weekly help with personal or household tasks for a parent who was unable to look after themselves.

#### 2.1.5 Health

At age 68–69, we captured the burden of disease by distinguishing participants with 0, 1, 2, 3+ doctor diagnosed diseases over the previous ten years. The research nurse asked the participant about 19 disorders: heart failure, angina, myocardial infarction, hyper/hypotension, stroke, diabetes, transient ischaemic attacks, cancer, chronic lung disease, asthma, osteoarthritis, rheumatoid arthritis, osteoporosis, serious eye trouble, depression, epilepsy, Parkinson’s disease, memory problems and kidney disease.

We also captured health-related limitations in six daily activities (walking ¼ mile, walking up and down stairs, difficulty keeping balance, bending down and straightening, reaching arms above head, and holding, gripping or turning something). Study members additionally reported longstanding illness that limited their usual activities at ages 68–69 and 60–64.

### 2.2 Statistical analysis

We first described expectations for those with and without a living child at age 68–69. Correlates of expectations for the sample with at least one child were then identified using multinomial regression models including i) gender only, ii) all covariates. Estimates are presented as average marginal effects, interpreted as the change in probability of the outcome per one unit change in exposure. (In preliminary analysis, we tested whether associations with any of the covariates were modified by gender and found no evidence for this, hence we present gender-adjusted rather than gender-stratified models.) For all analyses, we restricted the sample to those with observed data on care expectations and used multiple imputation by chained equations to impute missing covariate data in 20 datasets, under the assumption that these were missing at random.

Participation at the home visit at age 68–69 was highest among those with a higher number of prior contacts with the study, those with better self-rated health, and those with non-limiting longstanding illness (compared with no longstanding illness and limiting long-standing illness) [19]. Of the 2149 who participated in the age 68–69 home visit, 2135 provided data on expectations for their future care and form the analytical sample for the current study. Fifteen of these were excluded because they nominated a son-daughter but did not have a live child at age 68–69.

## 3 Results

Over 90% of study members at age 68–69 ha d an adult child and 44% of them lived less than 5 miles from their nearest adult offspring (Table 1). Almost 70% were married (either in their first or subsequent marriage). Over 40% had provided care for someone frail or with a disability between age 60 and 69, 3% provided this for 20 or more hours a week. Just under 50% had at least one functional limitation and 14% had a longstanding illness that limited their usual activities since age 60–64 or younger.

**Table 1:** Characteristics of the analytical sample (with complete data on expectations for future care).

	Total (N = 2120) %	Men (N = 1039) %	Women (N = 1081) %	P for gender difference
***Family factors***				
Geographical proximity to nearest adult child 68–69y				0.07
No adult child	8.9	9.4	8.4	
Overseas	1.9	2.2	1.7	
>100 miles	8.5	9.2	7.8	
25–100 miles	11.0	11.2	10.7	
5–24 miles	17.5	16.6	18.4	
1–4 miles	22.7	22.5	22.9	
<1 mile	15.7	13.3	18.0	
In same household	5.6	6.4	4.9	

Marital history up to 68-69y				<0.001
Never married	2.6	2.7	2.6	
In first marriage	55.5	59.9	51.3	
Remarried	14.3	14.6	13.9	
Separated/divorced	10.6	8.7	12.4	
Widowed	7.6	3.9	11.1	

***Socioeconomic position***				
Education 26y				<0.001
Below O-level	36.8	35.6	38.0	
O-level or equivalent	19.6	14.0	25.0	
A-level or equivalent	27.6	29.3	26.0	
Degree level	10.8	15.8	6.1	

Head of household social class 53y				<0.001
I/II (more advantaged)	47.7	55.9	39.9	
IIINM	23.9	10.3	37.0	
IIIM	15.1	24.3	6.2	
IV/V (less advantaged)	12.7	8.5	16.6	

***Characteristics of the social network***				
Frequency visit relatives & friends 60–64 & 68–69y; mean (sd)	10.3 (2.7)	9.96 (2.7)	10.75 (2.6)	<0.001
Help in a crisis 43, 53 & 60–64y				
Low	2.5	2.9	2.2	0.02
Medium	11.9	14.1	10.1	
High	85.6	83.1	87.8	

Emotionally close to parents 43y				0.07
Close to one or both	63.3	60.2	66.3	
Not close to either	14.9	16.0	14.0	
Not applicable	16.4	17.1	15.6	

***Care-giving experience***				
Looking after frail/disabled person 60–64 & 60–69y				0.6
No	57.9	58.6	57.4	
Giving some care	38.9	38.7	39.1	
Giving 20+ hrs/week at one or both ages	3.2	2.8	3.6	

Care of own parents when SM 43y				0.04
No care needed	86.3	86.2	86.4	
Care needed but SM did not provide	5.3	5.3	5.2	
SM provided care	2.3	1.4	3.1	

***Health***				
Number of chronic conditions 68-69y				0.3
0	25.1	26.8	23.5	
1	34.3	34.2	34.3	
2	20.0	19.2	20.8	
3+	20.6	19.8	21.3	

Number of functional limitations 68-69y				<0.001
0	51.4	64.7	38.6	
1	25.9	19.9	31.6	
2	9.8	6.1	13.4	
3	5.9	4.0	7.8	
4–6	7.0	5.3	8.6	

Limiting longstanding illness 60-64 & 68-69y				0.06
No	64.8	67.6	62.2	
At one age	21.3	19.2	23.1	
At both ages	14.0	13.2	14.7	

Almost 75% of study members with a living child expected to be cared for by their daughter or son in the event that they (and their spouse) needed help because of sickness, frailty or disability (Table 2). Whilst 11% of those with a living child expected to be cared for by paid professionals, this figure rose to 33% of those with no living child. Over 40% of those with no living child expected their future care to be provided by a friend or neighbour. There were no statistically significant gender differences in expectations for care.

**Table 2:** Expectations for future care for those with and without a living child at age.68–69.

	Has a living child (N = 1945)			Does not have a living child (N = 175)		
Expect care to be provided by	Total (N = 1945) %	Men (N = 947) %	Women (N = 998) %	Total (N = 175) %	Men (N = 92) %	Women (N = 83) %
Daughter/son	73.8	73.9	73.8	0.0	0.0	0.0
Other relative	49	5.8	4.0	25.1	26.1	24.1
Friend/neighbour	10.1	9.9	10.2	41.7	37.0	47.0
Paid professional/ no-one	11.2	10.4	12.0	33.1	37.0	28.9

The remainder of the analysis was based on those with a living adult child at age 68–69. Due to small numbers of never married participants with a living child (n = 4), this group was also dropped from the remainder of the analysis. Gender-adjusted models are summarised first. As anticipated, geographical proximity was strongly associated with expectations. Those living 25–100 miles away from the nearest child had 23.5% lower probability of expecting care from a daughter or son than those living within 5–25 miles (Table 3), 16.1% higher probability of expecting care from a friend/neighbour and a 6.4% higher probability of expecting care from a paid professional/no-one. Being separated/divorced was associated with lower probability of expecting care from a daughter or son and higher probability of expecting care from a friend/neighbour or paid professional compared with those who were married. Remarried participants had 8.4% lower probability of expecting care from a daughter or son compared with continually married participants. Socioeconomic advantage, whether captured by education or by occupational social class, was associated with lower probability of expecting care from a daughter or son and higher of expecting a friend/neighbour to provide future care.

**Table 3:** Social and health correlates of expectations for future care based on n = 1941 participants with a living child. Estimates are average marginal effects.

	From daughter or son		From other family		From friend/neighbour		From paid profession/no-one nominated	
	Gender adjusted	Fully adjusted^b^	Gender adjusted	Fully adjusted^b^	Gender adjusted	Fully adjusted^b^	Gender adjusted	Fully adjusted^b^
Female (vs male)	−0.2	−2.0	−1.9	**−2.9**	0.4	1.0	1.7	**3.6**
***Family factors***								
Geographical proximity to nearest adult child 68-69Y								
Overseas/100+ miles	**−44.0**	**−41.0**	**6.5**	**9.1**	**24.9**	**23.2**	**12.6**	**8.7**
25–100 miles	**−23.5**	**−21.**	1.0	1.8	**16.1**	**14.8**	**6.4**	4.3
5–25 miles	Ref	Ref	Ref	Ref	Ref	Ref	Ref	Ref
<5 miles including in same household	**14.6**	**13.6**	−1.8	−2.0	**−5.2**	**−5.0**	**−7.5**	**−6.5**
Marital history to 68-69y^a^								
Continually married	Ref	Ref	Ref	Ref	Ref	Ref	Ref	Ref
Remarried	**−8.4**	−3.8	0.9	0.0.3	3.6	1.5	3.8	2.0
Separated/divorced	**−17.9**	**−10.5**	4.0	2.6	**8.2**	**4.9**	**5.6**	3.0
Widowed	−6.7	**−8.4**	**5.9**	**5.9**	3.4	4.5	−2.5	−2.0

***Socioeconomic position***								
Education age 26 per 1 level increase	**−3.8**	0.4	−0.9	**−1.8**	**2.4**	1.7	**2.2**	1.2
Head of household social class age 53 per 1 level increase	**−3.3**	−0.4	2.2	0.5	**1.9**	−1.2	1.2	−0.2

***Characteristics of the social network***								
Frequency visit friends & relatives 60-69y								
Low	****−27.0****	****−7.6****	0.2	−1.8	****12.1****	1.7	****14.6****	****7.6****
Medium	**−11.0**	−3.4	2.1	1.0	**3.4**	−1.2	**5.5**	3.6
High	Ref	Ref	Ref	Ref	Ref	Ref	Ref	Ref
Help in crisis age 43-64y								
Never	**−21.2**	−10.3	1.3	1.6	2.9	−1.0	**17.0**	9.6
Sometimes	**−10.1**	**−6.1**	1.0	0.9	0.1	−1.6	**9.1**	**6.8**
Often/always	Ref	Ref	Ref	Ref	Ref	Ref	Ref	Ref
Emotionally close to parents 43y								
Not close to either	**−9.3**	**−5.0**	−1.2	−1.2	**5.2**	3.3	**8.2**	3.0
Not applicable	2.4	−1.2	−1.6	−0.9	−1.2	0.6	0.3	1.6
Close to at least 1 parent	Ref	Ref	Ref	Ref	Ref	Ref	Ref	Ref

***Care-giving experience***								
Looking after frail person 60-69y	Ref	Ref	Ref	Ref	Ref	Ref	Ref	Ref
No	−1.4	−5.8	0.2	0.0	0.3	−0.4	0.9	0.9
Giving some care Giving care 20+ hrs/wk	−4.8	−4.2	0.2	0.3	−0.9	−1.0	5.6	4.9
Care of own parents 43y								
No care needed	Ref	Ref	Ref	Ref	Ref	Ref	Ref	Ref
SM did not provide care	**−11.5**	−4.9	2.0	2.2	7.1	3.0	2.1	−0.3
SM provided care	−8.3	−8.8	3.0	2.1	0.4	0.9	5.0	5.9

***Health***								
Limiting longstanding illness 60-69y								
No	Ref	Ref	Ref	Ref	Ref	Ref	Ref	Ref
At one age	−1.7	−2.1	−1.8	−1.7	0.9	1.5	2.5	2.3
At both ages	3.4	3.6	−2.4	**−2.5**	−2.4	−1.6	1.3	0.5

**Bold** indicates p < 0.05.

a Never married study members were excluded from analysis due to small numbers with living children.

b Includes all variables listed in the Table.

Several social network characteristics were associated with expectations. Those with low compared with high frequency of contact with friends and relatives had 27.0% lower probability of expecting future care from a daughter or son and 14.6% higher probability of expecting future care to be provided by a paid professional. Not being able to rely on someone for help in a crisis between the ages of 43 to 60–64 and not being emotionally close to a parent at age 43 were also associated with higher probability of expecting a paid professional to meet any future care needs.

Care-giving experiences were not strongly associated with care expectations although those who did not provide care for their own infirm parents at age 43 had 11.5% lower probability of nominating a daughter or son compared with those whose parents did not need care at that age. In addition, those giving 20 or more hours care per week in their sixties had a 5.6% higher probability of nominating a paid professional to provide for their own future care needs (though this difference was not statistically significant).

Number of chronic conditions, functional limitations and longstanding illness were not strongly associated with care expectations (Supplementary Table 1). For this reason, and because of possible collinearity between the health indicators (with a polychoric correlation coefficient for health-related limitations and longstanding illness of 0.66), we included only longstanding illness at ages 60–64 and 68–69 in the main analysis.

Several of these correlates were inter-related and the associations described above were attenuated on mutual adjustment. Expectations did not differ by socioeconomic factors in the multiply-adjusted analysis and subsidiary analysis indicated that socioeconomic differences in expectations were primarily explained by geographical proximity to adult children. A total of 65% of those with low educational attainment lived within 5 miles of an adult child compared with 30% of those with a degree level qualification (Supplementary Table 2). However, the lower probability of expecting future care from a daughter or son remained among those who lived further away from their children, were separated/divorced, had low social contact, had long-term lack of someone to help in a crisis, and had low emotional closeness to their own parents in mid-adulthood. Independent of other covariates, a higher probability of expecting a paid professional to provide for future care needs remained among those who lived further away from their children, had low social contact, and had long-term lack of someone to help in a crisis.

## 4 Discussion

We examined who, besides the partner or spouse, would be expected to provide for the future care needs of a population-based sample of baby-boomers approaching their seventies. People may be optimistic about the extent to which they will receive support when it is needed [22]; others have suggested that expectations may lie between preferences and actual availability [20]. Almost three in four of those who had a living child expected them to meet their future care needs, but we also found that one in five of those with a living child and three in four of those without a living child expected their future needs to be met by a friend/neighbour or a paid professional. Marital history and proximity to adult children were strongly associated with expectations but, independently of these factors, aspects of the social network through mid to later adulthood were associated with who was expected to provide care. In particular, those who lacked contact with friends and relatives and those who did not earlier have someone to rely on in a crisis were relatively more likely to nominate a paid professional. Chronic conditions and functional limitations, on the other hand, were not related to expectations.

In contrast to most studies which have focused on preferences, we considered expectations for provision of future care needs. The expectation that adult children will provide care is in line with current figures on who actually provides care. Among older people currently receiving personal care, adult children, along with spouses, are the main providers [4]. Almost 45% of participants in this study were living within five miles of an adult child, a factor which facilitates informal care from adult children [23]. Nevertheless, we do not know whether the nominated children would be willing or able to provide care. A study based in the United States found parents in the United States anticipated needing more help from their adult children than their children anticipated them needing [24].

One in ten participants nominated instead a friend or neighbour. As expected, this was relatively more likely among those who had frequent contact with their friends. Whilst this higher contact might make it more likely that a level of support is forthcoming, it has been noted that friends and neighbours tend to provide emotional support and some tangible support [25] but less frequently provide help with tasks which are intensive or include personal care [26]. This group may therefore be overly optimistic about the care they will receive from friends and neighbours and may be at risk of not having any future care needs fully met informally.

One in ten participants nominated formal care, rising to one in three among those who did not have a living child. The proportion is in line with earlier estimates showing around one in ten men and one in five women aged 75 and over who have an activity limitation receive some formal care [27,28]. We did not collect information on whether participants expected the cost of this formal care to be met from their own funds or funded by the state, but the number of over 65 s in Britain receiving state-funded care has been falling steadily over the last decade [4]. In gender-adjusted analyses, socioeconomic advantage was associated with a higher probability of nominating a professional to meet future care needs, as has been found in other studies [[9], [10], [11]]. Although this may partly be due to costs, in our study this was explained by the greater geographical proximity of more socioeconomically disadvantaged participants to their adult children. Given increasing levels of educational attainment as well as increasing residential mobility across successive generations, expectations to rely on formal care might increase in future years. Geographical proximity may also indicate greater emotional closeness for some families. In our study, lack of emotional closeness to own parents in mid-adulthood and lack of someone to help in a crisis earlier in adulthood was associated with lower expectation that a person will rely on their own children for any future care needs. This illustrates how the receipt of personal care in later life is an integral part of multiple forms of care-giving and receiving in social relationships across the life course [16,29].

Experiencing divorce was associated with lower probability of nominating an adult child to provide personal care compared with those whose first marriage remained intact. In gender-adjusted analysis this was seen for those who remarried as well as those who did not re-partner. Other studies also found that unmarried people have lower expectations for help from adult children [9,20] and lower intergenerational support in later life [30] especially for fathers [29] though some studies did not find lower levels of support from adult children among parents who had been divorced [13,31]. The association we found between divorced status and expectations was partly attenuated by adjustment for frequency of contact with family and friends and closeness to own parents, as expected given evidence that marital dissolution can reduce the quantity or quality of family ties [30,32]. This further illustrates the role of social connectedness across the life course and across generations for later life care, and raises concerns about the implications of the rise in divorce rates that we have seen in recent decades.

We considered multiple health indicators based on doctor diagnosed conditions, functional limitations and limiting illness over the previous ten years. None of these was strongly related to expectations, a finding which stands in contrast to our hypothesis that long-term and severe illness would be associated with a greater expectation that care needs would be met formally. Possibly we had insufficient numbers of severely limited participants in this population-based sample, though others also found no association between current health needs and preferences [9].

Methodological strengths of the study include a large sample size, a population-based sampling frame, high response rates at all sweeps, and lack of confounding by age. We included prospective measures of socioeconomic position, characteristics of the social network, care-giving experience and health from earlier adulthood. Limitations of the study should be noted. We did not allow participants to select more than one source to provide for their future care needs. A previous study which allowed multiple sources (up to a maximum of four) to be nominated found mean number of expected caregivers was 1.7 [20]. That study allowed self and spouse to be nominated, however, and these options were commonly selected so our restriction may not have missed key sources for most participants. However, this restriction also meant that we did not allow mixed informal plus formal support as an option, in line with the hierarchical model of care, which has empirical support [20]. Nevertheless, mixed formal care and informal care (in line with the complementary model of care [33]) is the preferred option for many [9]. We did not specify whether the help would be needed over the short or long-term. Nor did we specify the tasks that help would be needed with and we acknowledge that preferences vary according to the nature of the condition and the care task [34]. We did not consider daughters and sons separately because we did not have full information on live adult children at this age, but having a daughter available locally has previously been associated with greater likelihood of expecting to rely on an adult child [20]. Attrition is inevitable after 70 years of follow-up, with socioeconomically disadvantaged and less healthy individuals more likely to not respond [35]. However, in our multiply-adjusted analyses, health and socioeconomic position were not related to expectations. We are not aware that other studies based in Britain have described expectations among this baby-boomer generation but we note that social care provision and preferences vary across nations with different social norms and social policies [[36], [37], [38]] and may not be generalizable to other nations or birth cohorts so replication in other settings would be valuable.

This study set out to describe people’s expectations for their future care provision at age 68–69. This is timely because from age 70 onwards, older people tend to be net receivers of support from their children but at younger ages they tend to be net support providers [39]. We focused on expected sources of personal care in the scenario that this is not available from the spouse. Lack of social relationships was linked to greater expectation to use formal care in later life. The strongest correlate was geographical distance which may partly be a consequence of emotional distance. Personal care is increasingly being provided informally by family members, especially spouses and adult children. Notwithstanding questions regarding the benefits to care recipients and providers of this strategy, if the trend is to continue then we need to tackle factors that might be a barrier or provide incentives for doing so. These findings suggest this might include reducing barriers to residential mobility in later life, such as the costs of moving home [40] so that geographical distances between parents and children can be reduced. It might also include greater support to build and maintain high quality family relationships across the life course.

## Contributors

Both authors planned the study, interpreted the results, edited the paper and approved the final version.

Mai Stafford conducted the statistical analysis and drafted the paper, and is guarantor accepting full responsibility for the work, had access to the data and controlled the decision to publish.

## Conflict of interest

The authors declare that they have no conflict of interest.

## Funding

This work was supported by the UK Medical Research Council [grant numbers MC_UU_12019/1; MC_UU_12019/2; MC_UU_12019/5].

The funders had no involvement in the collection, analysis or interpretation of data or the writing of the report.

## Ethical approval

This study abided by the human rights code of ethics and guidelines. Ethical approval was obtained from the NRES Queen Square REC (14/LO/1073) and Scotland A REC (14/SS/1009).

## Provenance and peer review

This article has undergone peer review.

## Research data (data sharing and collaboration)

Data used in this publication are available to bona fide researchers upon request to the NSHD Data Sharing Committee via a standard application procedure. Further details can be found at http://www.nshd.mrc.ac.uk/data. doi: https://doi.org/10.5522/NSHD/Q102; https://doi.org/10.5522/NSHD/Q103.
