# Supplementary material for: Expectations for future care provision in a population-based cohort of baby-boomers
Source: Maturitas. 2018 Oct;116:116–22. doi: 10.1016/j.maturitas.2018.08.004 (PMC6189454; doi:10.1016/j.maturitas.2018.08.004)
Supplement: Supplementary file 1 [file mmc1.docx]

Supplementary Table 1. Alternative health indicators and care expectations. Estimates are average marginal effects.

|  | **Expect to be cared for by daughter or son** | **Expect to be cared for by other family** | **Expect to be cared for by friend/neighbour** | **Expect to be cared for by paid professional/no-one nominated** |
| --- | --- | --- | --- | --- |
|  |  |  |  |  |
| ***Health*** |  |  |  |  |
| Number of chronic conditions age 68-69  0  1  2  3+  Functional limitations age 68-69  0  1  2  3  4+  Limiting longstanding illness age 60-64 & 68-69  No  At one age  At both ages | Ref  -1.6  -4.5  -0.6  Ref  0.3  -4.0  -2.0  0.0  Ref  -1.7  3.4 | Ref  0.0.2  1.3  1.8  Ref  0.8  0.2  1.0  1.4  Ref  -1.8  -2.4 | Ref  -0.0.2  -0.0  -2.3  Ref  -1.5  -2.0  -0.1  -1.8  Ref  0.9  -2.4 | Ref  1.6  3.3  1.1  Ref  0.4  **6.2**  1.6  0.3  Ref  2.5  1.3 |

Supplementary Table 2. Educational attainment and geographical proximity to adult child

|  | Educational attainment | | | |
| --- | --- | --- | --- | --- |
| Geographical proximity | Below O-level (lowest)  % | O-level or equivalent  % | A-level or equivalent  % | Degree level (highest)  % |
| Overseas/100+ miles | 7.4 | 12.4 | 13.1 | 25.0 |
| 25-100 miles | 8.8 | 11.0 | 17.3 | 21.9 |
| 5-25 miles | 19.1 | 20.5 | 24.6 | 23.4 |
| <5 miles including in  same household | 64.8 | 56.2 | 45.0 | 29.7 |
